# MyD88 signaling pathways: role in breast cancer

**DOI:** 10.3389/fonc.2024.1336696

**Published:** 2024-01-29

**Authors:** Hongmei Zheng, Xinhong Wu, Liantao Guo, Jianhua Liu

**Affiliations:** ^1^ Department of Breast Surgery, Hubei Cancer Hospital, Tongji Medical College, Huazhong University of Science and Technology, Hubei Provincial Clinical Research Center for Breast Cancer, Wuhan Clinical Research Center for Breast Cancer, Wuhan, Hubei, China; ^2^ Department of Breast and Thyroid Surgery, Renmin Hospital of Wuhan University, Wuhan, China

**Keywords:** breast cancer, myeloid differentiation factor 88, epithelial-mesenchymal transition, tumor microenvironment, biomarker

## Abstract

MyD88 plays a central role in breast cancer, exerting a multitude of effects that carry substantial implications. Elevated MyD88 expression is closely associated with aggressive tumor characteristics, suggesting its potential as a valuable prognostic marker and therapeutic target. MyD88 exerts influence over several critical aspects of breast cancer, including metastasis, recurrence, drug resistance, and the regulation of cancer stem cell properties. Furthermore, MyD88 modulates the release of inflammatory and chemotactic factors, thereby shaping the tumor’s immune microenvironment. Its role in immune response modulation underscores its potential in influencing the dynamic interplay between tumors and the immune system. MyD88 primarily exerts intricate effects on tumor progression through pathways such as Phosphoinositide 3-kinases/Protein kinase B (PI3K/Akt), Toll-like Receptor/Nuclear Factor Kappa B (TLR/NF-κB), and others. Nevertheless, in-depth research is essential to unveil the precise mechanisms underlying the diverse roles of MyD88 in breast cancer. The translation of these findings into clinical applications holds great promise for advancing precision medicine approaches for breast cancer patients, ultimately enhancing prognosis and enabling the development of more effective therapeutic strategies.

## Introduction

Breast cancer (BC) is one of the most prevalent malignant tumors in women (1). The global burden of breast cancer annually amounts to over 2.2 million diagnosed cases, resulting in over 600,000 fatalities (2). Encouragingly, in high-income countries, there has been a decline in breast cancer mortality, largely attributed to advancements in treatment modalities. However, there’s a steady rise in breast cancer incidence, and it remains the most common cause of cancer-related deaths in low-income countries (3). Clinically, specific subtypes of breast cancer are characterized by their histological appearance and the expression of hormone receptors and growth factors, namely estrogen receptor (ER), progesterone receptor (PR), and human epidermal growth factor receptor 2 (HER2) (4). Characterized by remarkable heterogeneity, breast cancer is primarily driven by factors such as metastasis, recurrence, and drug resistance, which contribute significantly to patient mortality (3).

Against this backdrop, Myeloid Differentiation Factor 88 (MyD88) assumes a crucial role in breast cancer. The MYD88 gene provides instructions for producing proteins involved in signaling within immune cells. Acting as an adapter, the MyD88 protein plays a crucial role in innate immunity by mediating cell activation through Toll-like receptors (TLRs). TLRs interact with adapter protein MyD88, initiating the activation of two key transcription factors: NF-κB, a dimeric protein responsible for expressing various inflammatory cytokines, chemokines, adhesion, and co-stimulatory molecules, triggering acute inflammation and stimulating the immune response. IRFs, a group of proteins responsible for expressing type I interferons, to establish the so-called antiviral state of cells (5). While MyD88 is renowned for the role in recognizing and responding to microbial pathogens in innate immunity (6, 7), it also plays a pivotal role in numerous non-immune processes, particularly within the context of tumor development. Currently, MyD88 is regarded as a critical signaling molecule with diverse roles in the development and progression of breast cancer. Engaging in various signaling pathways, MyD88 influences proliferation, migration, and invasion of breast cancer cells. Through cascading responses such as Phosphoinositide 3-kinases/Protein kinase B (PI3K/Akt), Wingless/Integrated (Wnt), Notch, Hedgehog, and Nuclear Factor kappa B (NF-κB), it orchestrates the epithelial-mesenchymal transition (EMT), conferring migratory and invasive traits upon tumor cells (8–15). By modulating signaling pathways governing the maintenance and functionality of breast cancer stem cells (BCSCs), MyD88 regulates tumor initiation, progression, recurrence, and therapy resistance (16). Activation of MyD88 intricately correlates with self-renewal, cytokine production and secretion, as well as expansion of tumor stem cells, thereby influencing tumor development and progression.

Through modulation of inflammatory responses and immune reactions, MyD88 impacts the immune microenvironment of breast cancer (17). It governs the effects of tumor cell-derived exosomes, stimulating the production of inflammatory factors and influencing the activity of macrophages, T cells, and NK cells. Moreover, the interaction of MyD88 with immune checkpoint therapy plays a pivotal role, significantly impacting breast cancer treatment outcomes. Notably, the expression level of MyD88 level closely associates with disease severity, prognosis, and staging of breast cancer (18). High MyD88 expression correlates intimately with clinical parameters like tumor size, lymph node metastasis status, and histological grade. Patients with high MyD88 expression tend to experience recurrences or metastases, whereas those with low expression exhibit better prognosis.

**Figure 1:**
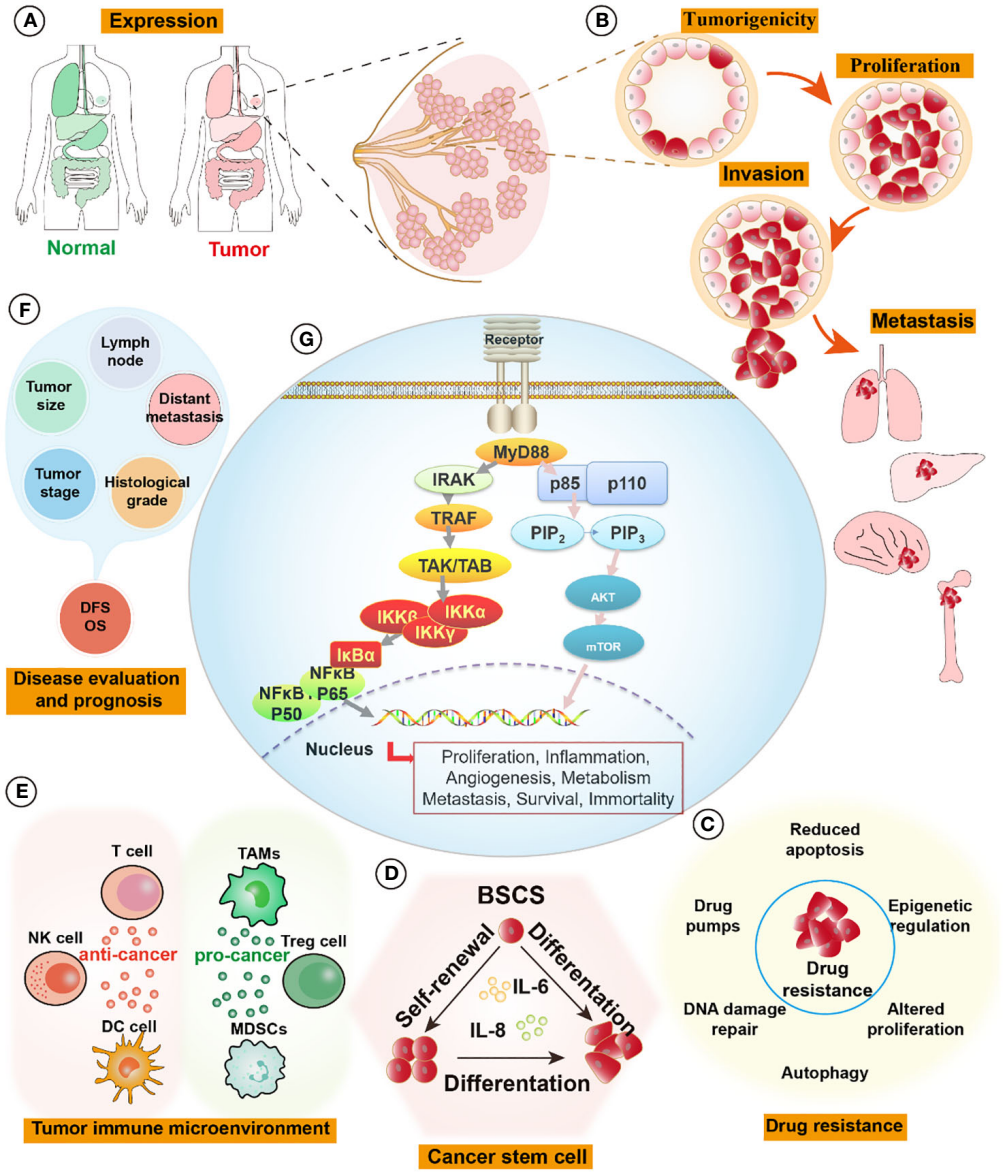
MyD88 signaling pathways in breast cancer. **(A)** The expression of MyD88 is elevated in tumor tissues compared to normal tissues, such as those found in breast, lung, liver, colon, and stomach organs. **(B)** Activation of the MyD88 pathway fosters the formation of cell spheroids and enhances tumor-forming capabilities. It influences tumor proliferation by activating cell cycle pathways and augments cell invasion and distant metastatic capabilities by activating relevant pathways like those associated with EMT signaling pathways. **(C)** MyD88 enhances tumor cell resistance to drugs by pumping drugs out, promoting DNA repair, inhibiting apoptosis, and activating cell survival pathways. **(D)** Upregulation of the MyD88 signaling pathway promotes self-renewal and differentiation of breast tumor stem cells, augmenting their survival capabilities. **(E)** In the tumor immune microenvironment, MyD88 exerts intricate effects. On one hand, it promotes tumor progression by increasing the infiltration and enrichment of tumor immunosuppressive cells (TAMs, Tregs, and MDSCs), thereby facilitating tumor growth and metastasis. On the other hand, it demonstrates anti-tumor effects by activating DC cells, NK cells, and tumor-killing T cells, initiating tumor-specific immunity and eliminating tumors. **(F)** The expression levels of MyD88 correlate significantly with breast tumor size, histological grading, lymph node involvement, tumor staging, distant metastasis, and overall prognosis. **(G)** MyD88 influences tumor proliferation, inflammation, angiogenesis, metabolism, metastasis, survival, and immortality through the Phosphoinositide 3-kinases/Protein kinase B (PI3K/Akt) and Toll-like Receptor/Nuclear Factor Kappa B (TLR/NF-κB) pathways. EMT, epithelial-mesenchymal transition; MDSCs, Myeloid-Derived Suppressor Cells, TAMs, Tumor-Associated Macrophages, Tregs, Regulatory T cells.

In this review, we provide a comprehensive overview of MyD88’s roles in tumorigenesis, metastasis, drug resistance, the tumor microenvironment, and prognosis in breast cancer (**Figure 1**). We also explore potential avenues through which this understanding can drive future drug development. By delving deeper into the multifaceted impacts of MyD88 in breast cancer, we anticipate the emergence of novel strategies for the treatment and management of this disease.

## The role of MyD88 in clinical disease assessment and prognosis

MyD88 assumes a pivotal role in the clinical evaluation and prognosis of breast cancer. A multitude of studies has revealed a profound association between MyD88 expression levels and the severity and prognostication of breast cancer (**Table 1**). Elevated MyD88 expression is correlated with larger tumor size, lymph node metastasis, and higher histological grades, suggesting MyD88’s potential as a biomarker for appraising breast cancer’s invasiveness and progression. The protein expression of MyD88 demonstrates a significant positive correlation with tumor size, stage, axillary lymph node metastasis, and distant metastasis (19). MyD88 protein expression is positively linked with axillary lymph node metastasis and histological grade. Both TLR4 and MyD88 protein expressions are positively correlated with breast cancer cell metastasis (18). Notably, MyD88 protein expression in breast cancer tissue surpasses that in adjacent non-cancerous tissue, and it is positively associated with axillary lymph node metastasis, histological grade, and distant metastasis (18). TLR4 and MyD88 protein expression levels are positively correlated with axillary lymph node metastasis and histological grade, and the co-expression of TLR4 and MyD88 is also positively correlated with breast cancer cell metastasis (18). The expression rate of MyD88 is notably higher in samples from patients with axillary lymph node metastasis (59%) compared to those without metastasis (25.6%). Similarly, the expression rate of MyD88 is higher in patients with stage III disease (65.6%) compared to those with stage I/II disease (44.1%) (18). The expression of MyD88 is intrinsically connected to patient prognosis, with higher MyD88 expression increasing the propensity for recurrence or metastasis. MyD88 expression levels inversely correlate with disease-free survival (DFS) and overall survival (OS), with lower MyD88 expression translating to better patient outcomes (21). A notable divergence in survival rates is evident between high and low MyD88 expression groups, as evidenced by Xiang’s study (20). Kaplan-Meier survival curves underscore the significance of MyD88 expression in survival disparities between patients with high MyD88 expression and those with normal expression (20). Furthermore, MyD88’s protein expression level exhibits a positive correlation with axillary lymph node metastasis and histological grade, while the co-expression of TLR4 and MyD88 is positively correlated with breast cancer cell metastasis (18).

**Table 1:** MyD88 expression and clinical evaluation in breast cancer.

Technology	Results	Ref.
IHC	The expression of MyD88 in malignant tumors significantly surpasses that in adjacent normal tissue and benign tumors.	(18–20)
	Significant differences are observed in DFS and OS curves concerning MyD88 expression levels. Patients with lower MyD88 expression exhibit better DFS and OS compared to those with higher expression levels. Multifactorial analysis reveals MyD88 status as an independent risk factor for DFS and OS.	(20, 21)
	MyD88 expression correlates with ER and PR statuses, and patients with higher MyD88 expression experience more frequent recurrence or metastasis.	(21)
	No correlation has been found between MyD88 expression and ER or PR status.	(20)
	MyD88 expression levels relate to breast cancer TNM staging, being more detectable in stage III compared to stage I or II breast cancer patients.	(20)
WB	High expression of MyD88 significantly correlates with tumor size, tumor staging, axillary lymph node metastasis, and distant metastasis.	(19)
	The protein expression level of MyD88 in MDA-MB-231 cells is 1.6 times that of MCF-7 cells and 1.8 times that of MDA-Kb2 cells.	(18)
	High levels of MyD88 protein expression were confirmed in hormone-resistant breast cancer cell lines MDA-MB-231, MDA-MB-231HM, and MDA-MB-468, whereas such confirmation was not observed in MCF-7 and SKBR3 cell lines.	(21)
qRT-PCR	The expression of the MyD88 gene in breast cancer tissues is higher compared to adjacent normal tissues and benign tumors.	(19)
	In MDA-MB-231 cells, the gene expression level of MyD88 is 2.09 times higher than in MCF-7 cells.	(18)
	Zandi et al. assessed the baseline expression levels of MyD88 in different breast cancer cell lines—MCF7, SKBR3, MDA-MB-231, and BT-474—and observed the highest MyD88 mRNA level in MCF-7 cells.	(22)
IF	The average fluorescence intensity of MyD88 in MDA-MB-231 and MCF-7 cells was 0.136 and 0.05, respectively, there was no significant difference between the levels in MCF-7 and MDA-Kb2 cells	(18)

DFS, Disease-Free Survival; IF, Immunofluorescence; IHC: Immunohistochemistry; OS, Overall Survival; qRT-PCR: Quantitative Reverse Transcription Polymerase Chain Reaction; WB, Western Blotting.

MyD88, therefore, emerges as a potential diagnostic and therapeutic target in breast cancer. The assessment of MyD88 expression levels offers a means to comprehensively gauge breast cancer invasiveness and predict patient prognosis, thereby establishing a foundation for informed decisions in personalized treatment.

## MyD88 expression in breast cancer

MyD88, a pivotal signaling molecule, has garnered attention for its dysregulated expression in various cancer types, including colorectal cancer (23), ovarian cancer (24), hepatocellular carcinoma (25), and pancreatic cancer (26), highlighting its role in tumor progression. In breast cancer, the expression of MyD88 varies within breast cancer cells, tumor tissues, and the surrounding microenvironment. While normal breast tissues typically maintain low MyD88 expression levels for physiological functions, breast cancer often exhibits significant upregulation. Studies indicate that adjacent normal tissues and benign breast tumors have lower MyD88 expression, whereas breast cancer demonstrates heightened and pronounced expression (19).

Differential MyD88 expression may also exist among distinct molecular subtypes of breast cancer. Zandi et al. conducted baseline expression level assessments of MyD88 in various breast cancer cell lines, including MCF7, SKBR3, MDA-MB-231, and BT-474, and found that MyD88 mRNA level was highest in MCF-7 cells (22, 27). However, another study revealed that within the MDA-MB-231 cell line, both the mRNA and protein expression levels of MyD88 were notably elevated in comparison to MCF-7 and MDA-Kb2 cells (which stably express an androgen- and glucocorticoid-responsive reporter) (18). Furthermore, there is evidence of a connection between MyD88 and tumor cell resistance. Ma et al. confirmed elevated levels of MyD88 protein in hormone-resistant breast cancer cell lines, MDA-MB-231, MDA-MB-231HM, and MDA-MB-468, compared to MCF-7 and SKBR3 cells (18). MyD88 and TLR4 expression and other clinical and pathological parameters. The expression of MyD88 was associated with ER and PR status (p<0.001). TLR4 expression was associated with PR status (p = 0.015) (21). In another study, it was demonstrated that there is no correlation between the expression of MyD88 and the status of ER or PR (20). X. Chen et al. found that heightened MyD88 expression is linked to increased malignancy and unfavorable prognosis in breast cancer. Elevated levels of MyD88 expression show a significant correlation with tumor size, staging, axillary lymph node metastasis, and distant metastasis (19).

In summary, MyD88 expression in breast cancer exhibits variability. Understanding the expression patterns of MyD88 contributes to a better comprehension of its roles in breast cancer initiation, progression, and treatment. This knowledge provides essential insights into its potential as a therapeutic target.

## Effect of MyD88 on tumorigenicity

The MyD88 protein exerts a profound influence on cancer cell tumorigenesis and the initiation of tumor growth. Serving as a crucial signaling adaptor protein, the TLR/MyD88 pathway plays a pivotal role in tumorigenicity. Activation of the MyD88 pathway promotes tumor formation, while MyD88 knockdown results in reduced clonogenicity in primary ER^neg^ tumors (14). Inhibiting MyD88 leads to decreased clonogenicity, and MyD88 shRNAs result in negative selection in the development of *in vivo* xenograft tumors derived from ER^neg^ breast cancer cells. Depletion of MyD88 in MCF-7 cells markedly inhibits tumor growth in nude mice, with tumor volumes generated from MyD88-deficient MCF-7 cells approximately half the size of those from control cells (20). TLR2, an upstream signaling molecule of MyD88, depletion impedes the tumor-initiating capacity of cells (28). For instance, TLR2-depleted spherical mammospheres cells only gave rise to tumor growth in 5 out of 14 mice, in contrast to control cells, which developed rapidly growing tumors in all mice (28). Additionally, Tlr2^-/-^MMTV–Wnt1 (Tlr2^-/-^Wnt1) mice exhibit markedly reduced tumor formation compared with that of their Tlr2^+/+^MMTV–Wnt1 (Tlr2^+/+^Wnt1) littermates, with median tumor-free days of 269 and 137, respectively (14). TLR2 neutralizing antibodies also block the growth of two independent ER^neg^ breast cancer xenografts *in vivo*. Short hairpin RNA (shRNA)-mediated knockdown of TLR2 in two breast cell lines (MDA-MB-468 and MDA-MB-231) similarly decreased clonogenic outgrowth (14). Moreover, suppression of constitutive downstream NF-κB activity in human breast cancer cell lines leads to reduced tumorigenicity (29). Furthermore, inhibiting NF-κB activity in human breast cancer cell lines can induce cell apoptosis (30) or diminish tumor-forming capability (31). Collectively, these findings underscore the significance of the TLR2/MyD88/NF-κB pathway in tumorigenicity. These observations imply that the TLR2/MyD88/NF-κB pathway is implicated in *de novo* mammary tumor formation.

## Effect of MyD88 on breast cancer proliferation

Cell proliferation is a central driver of breast cancer development and progression (32). In breast cancer, MyD88 is recognized as a key regulator with a significant impact on cell proliferation. Our previous studies have demonstrated that lipopolysaccharide (LPS) activates the MyD88 signaling pathway, promoting the *in vitro* proliferation of MCF-7 and MDA-MB-231 cells, as well as enhancing growth in xenograft mouse models *in vivo*. In our preliminary studies, a novel compound (TJ-M2010) was synthesized, drawing inspiration from the MyD88 molecular structure. This compound was engineered to specifically bind to the TIR domain of MyD88, aiming to disrupt the dimerization process of MyD88 (11). The application of TJ-M2010 hinders MyD88/NF-κB signaling transduction, resulting in the suppression of breast cancer cell proliferation mentioned above (33). Liu et al. employed a small-molecule compound known as 4210, which obstructs MyD88 dimerization (34), Their findings suggested that 4210 inhibition of MyD88 might induce nonspecific cell death in MDA-MB-231 cells (35). Another study demonstrated that treatment using the MyD88 inhibitor (ST2825) resulted in reduced growth of murine mammary carcinomas 4T1, 168, EMT6, and SM1, as well as the human breast cancer cell line MDA-MB-231 (36). In another study, a TLR3 ligand notably increased breast cancer cell proliferation, as indicated by the upregulation of cyclin D1 expression. However, the introduction of a MyD88 inhibitor disrupted the signal transduction of the TLR3-MyD88-NF-κB (p65)-IL6-Cyclin D1 pathway, leading to reduced breast cancer cell proliferation (37). Holleman et al. also confirmed that reduced MyD88 expression significantly inhibited MCF-7 cell proliferation (38). Similarly, the silencing of the MyD88 gene resulted in an increased G2 phase cell population in MCF-7 cells (20). NF-κB, a canonical downstream signaling molecule of TLR/MyD88, stimulates the transcription of the cyclin D1 promoter, thereby promoting cell growth (39). Additionally, caffeic acid phenethyl ester inhibited MDA-MB-231 cell proliferation by suppressing the TLR4/MyD88/NF-κB signaling pathway (40). Furthermore, MyD88 can affect breast cancer proliferation through the PI3K/AKT signaling pathway. The interaction between MyD88 and p85 enhances tumorigenic capabilities through the PI3K/Akt pathway (15). The MyD88 inhibitor TJ-M2010 interferes with the MyD88/PI3K/GSK-3β axis, consequently restraining breast cancer cell proliferation (33).

In conclusion, MyD88 plays a pivotal role in regulating breast cancer cell proliferation through the activation of multiple key signaling pathways. This highlights MyD88 as a significant potential therapeutic target, and inhibiting its function may contribute to restraining the growth and dissemination of breast cancer cells. However, further research is needed to gain deeper insights into the regulatory mechanisms of MyD88 in breast cancer cell proliferation, with the goal of developing more precise and effective treatment strategies.

## The effect of MyD88 on metastasis

Metastasis represents a pivotal stage in cancer progression, involving the spread of cancer cells from the primary tumor site to distant organs or tissues, where secondary tumors form (41). MyD88 is a molecular orchestrator with multifaceted implications for cellular processes central to cancer metastasis. Multiple studies have underscored the substantial involvement of MyD88 in tumor metastasis. For example, ectopic expression of MyD88 has been shown to enhance the invasion of hepatocellular carcinoma cells, whereas MyD88 inhibition has led to reduced lung metastasis and portal vein tumor thrombosis (15, 25, 42). MyD88 also plays a critical role in breast cancer metastasis. K. Wu et al. found that MyD88 expression was increased in highly invasive BC cell lines compared to low invasive BC cell lines, suggesting that MyD88 could be a potential therapeutic target for the metastasis of BC (18). Research has indicated that the heightened downstream NF-κB activity resulting from MyD88 elevation leads to increased expression of invasion-associated proteases (43, 44). Moreover, NF-κB contributes to the induction and maintenance of epithelial-mesenchymal transition (EMT) in Ras-transformed breast epithelial cells, mediating invasive and metastatic tumor phenotypes. Substantial evidence supports the role of NF-κB in late-stage breast tumor development and metastasis (45). In MDA-MB-231 cells, the downregulation of MyD88 influences NF-κB nuclear translocation, resulting in a diminished invasion capacity of tumor cells (36). Additionally, by knocking down MyD88, the migration speed of MCF-7 cells is reduced, and their ability to traverse Matrigel is impaired. In another study, tumors with reduced Myd88 levels displayed reduced growth and metastasis (46). MiR-4317 inhibits the proliferation, migration, and invasion of breast cancer cells by targeting MyD88 (47). Advanced glycation end products (AGEs) can promote breast cancer cell migration and invasion through the activation of the RAGE/TLR4/MyD88 signaling cascade (48). Berberine significantly inhibits the TLR9-MyD88-NF-κB pathway, reversing breast cancer metastasis (49). LPS enhances invasiveness and metastatic potential of breast cancer cells by upregulating the MyD88-BLT2-NF-κB signaling cascade (50). MCF-7 cell lines, after 48 hours of incubation with 0.5 µM morphine, exhibit downregulated TLR4/MyD88/NF-κB pathways, resulting in decreased migration (51, 52). TLR4 silencing reduces the expression of MyD88 and MMP9, significantly inhibiting cell migration and invasion (53). Furthermore, a TLR4 antagonist blocks TLR4 and MyD88 expression, as well as cell invasion and migration (54).

EMT, which refers to the transformation of epithelial cells into mesenchymal-like cells, conferring migratory and invasive properties (55), is a pivotal process in cancer. Various cascading reactions relevant to cancer have been established as critical regulatory signals of EMT (56, 57). The MyD88/NF-κB signaling pathway plays a crucial role in Snail-mediated EMT (58). EpRas cells are oncogenic and completely polarized Ha-Ras-transformed EpH4 mammary epithelial cells that undergo EMT, leading to the generation of mesenchyme-like cells (EpRasXT cells). Blocking the MyD88/NF-κB pathway eliminates the metastatic capability of mammary epithelial cells in murine models. Additionally, the reversal of NF-κB activity in EpRasXT cells nullifies the EMT process (45). Curcumin inhibits LPS-induced EMT in MDA-MB-231 and MCF-7 cells through the TLR4/MyD88/NF-κB/Snail signaling pathway (59, 60). The MyD88 inhibitor TJ-M2010-2 reverses TGF-β1-induced HK-2 cell EMT (61, 62). Similarly, the MyD88 inhibitor TJ-M2010-2 suppresses the proliferation, migration, and invasion of breast cancer cells by intervening in the MyD88/GSK-3β and MyD88/NF-κB signaling pathways (33).

In conclusion, MyD88 promotes cancer cell migration, invasion, and the establishment of secondary tumors in distant organs through diverse pathways, significantly impacting breast cancer metastasis. A comprehensive understanding of MyD88’s mechanisms in metastasis aids in devising effective strategies to inhibit cancer spread, thereby enhancing patient prognosis.

## The effect of MyD88 on drug resistance

Drug resistance poses a formidable challenge in cancer therapy, frequently resulting in reduced treatment effectiveness and disease relapse (63). MyD88 exerts a significant impact on drug resistance in breast cancer cells and is recognized as a key player associated with paclitaxel (PTX) resistance. Xiang et al. demonstrated the reversal of PTX resistance in breast cancer cells by targeting the miRNA-149-5p/MyD88 axis using ursolic acid (UA) (8). The PI3K/Akt pathway, a pivotal signaling route, becomes activated in PTX-resistant breast cancer (64). The observed heightened sensitivity of MCF-7 cells to paclitaxel following MyD88 downregulation implies its impact on inhibiting NF-κB activation, thereby blocking the PI3K/Akt signaling pathway (20). Elevated MyD88 levels in MDA-MB-231/PTX-resistant cells correlated with enhanced resistance, but MyD88 silencing increased PTX sensitivity (8). Activation of Akt may be linked to reduced paclitaxel-induced apoptosis (20), and MyD88 knockdown significantly suppressed Akt pathway activation in 231/PTX cells (8). The MyD88-regulated PI3K/Akt pathway appeared to mediate paclitaxel resistance in breast cancer cells by modulating Bax/Bcl-2 expression (8). Overexpression of MyD88 protein was associated with acquired paclitaxel resistance in triple-negative breast cancer (TNBC) cells. Dimethoxy-substituted compounds significantly reduced MyD88 overexpression in TLR4^+^ MDA-MB-231 cells and enhanced PTX activity (65). *In vitro* and *in vivo* experiments reversed paclitaxel resistance by inhibiting the target gene MyD88 (66).

Furthermore, the MyD88/NF-κB signaling pathway plays a central role in drug resistance in breast cancer. Downregulation of MyD88 increased sensitivity to paclitaxel in MCF-7 cells by suppressing NF-κB activation and blocking the PI3K/Akt pathway (20). The NF-κB/Notch1 regulatory loop contributes to the maintenance of breast cancer stem cells and drug resistance (16). Additionally, the NF-κB pathway promotes the transcription of ABCC1/MRP1 and ABCG2/BCRP transporters (67). This pro-survival signaling pathway becomes more active in response to radiotherapy, chemotherapy, and trastuzumab in breast cancer cells (68–70). Consequently, the MyD88/NF-κB pathway is involved in mediating drug resistance mechanisms in tumor cells. Despite substantial evidence supporting the regulatory role of MyD88 in drug resistance, some studies have yielded contrasting results, such as LPS pre-treatment sensitizing MyD88-positive cells to docetaxel cytotoxicity. This suggests that pathways induced by LPS stimulation could serve as attractive targets for overcoming therapy-resistant diseases (71).

A comprehensive understanding of MyD88’s role in drug resistance is crucial for devising strategies to overcome resistance and enhance the effectiveness of cancer treatment. Targeting MyD88 or its associated signaling pathways might offer a potential approach to increase cancer cell sensitivity to treatment and improve therapeutic outcomes. Further research is required to elucidate the potential mechanisms underlying MyD88-mediated drug resistance in breast cancer and other cancer types, as well as to explore therapeutic intervention methods.

## The effect of MyD88 on cancer stem cells

Breast cancer stem cells (BCSCs) constitute a small subset of tumor cells endowed with the unique capacity for self-renewal and differentiation into diverse cell types (72). These cells wield a pivotal role in tumor initiation, progression, recurrence, and resistance to therapy.

MyD88 has been revealed as a regulator of the characteristics and behavior of BCSCs through diverse mechanisms, actively participating in signaling pathways that govern the maintenance and functions of BCSCs. For instance, Conti et al. observed that the stimulation of TLR2 with ligands like Pam3CSK4, LTA, and PGN-SA significantly enhances mammosphere formation. In contrast, the action of the MyD88 homodimerization-inhibitory peptide markedly hinders the formation of spherical mammospheres (28). Silencing the TLR2/MyD88 signaling pathway also reduces mammosphere generation in both mouse (4T1) and human (MDA-MB-231, MCF7) mammary cells (28). The upregulation of the MyD88/NF-κB signaling pathway acts as a pro-survival route implicated in the self-renewal of BCSCs (16). The NF-κB protein p65 directly binds to the Gli1 promoter, enhancing its transcription (73). Additionally, the MyD88/NF-κB signaling pathway governs the expression of various cytokines, notably IL-6 and IL-8, which are intricately linked to tumor progression and the survival of BCSCs (74, 75). Extracellular IL-6 induces malignant characteristics in ductal breast cancer stem/progenitor cells (74), while the overexpression of IL-8 promotes stemness, stromal traits, acquisition of resistance, and the recruitment of immunosuppressive cells that foster tumor growth in the tumor microenvironment (75). Novel therapies targeting IL-8 receptor signaling may serve as a means to halt tumor progression. Machilin D reduces the secretion of IL-6/IL-8 and inhibits sphere growth (76).

In conclusion, MyD88 plays a significant role in regulating breast cancer stem cell characteristics and functions. A more profound understanding of the mechanisms through which MyD88 influences BCSCs holds the potential for the development of targeted therapies against these aggressive tumor-initiating cells. This offers both theoretical and practical implications for enhancing breast cancer treatment outcomes. Further research is warranted to comprehensively elucidate the mechanisms of MyD88 in BCSCs and its potential value as a therapeutic target.

## The influence of MyD88 on the tumor immune microenvironment

The influence of MyD88 on the tumor immune microenvironment is of great significance in the context of breast cancer progression. The tumor microenvironment is a complex ecosystem comprising tumor cells and their immediate surroundings, exercising a profound impact on tumor development (77–79). Through the modulation of inflammatory responses and immune reactions, MyD88 orchestrates changes within the tumor immune microenvironment. Exosomes released by tumor cells can activate the TLR/MyD88/NF-κB signaling pathway in macrophages, prompting the release of pro-inflammatory cytokines (such as IL-6, TNF-α, GCSF, and CCL2) (80). Within breast cancer stem cells, the HMGB1/TLR2/MyD88 axis governs NF-κB activation, IL-6 and TGF-β production, and the activation of STAT3 and Smad3. IL-6, stimulated through STAT3, fosters malignant properties, expanding the breast cancer stem cell population (81). The TGF-β signaling pathway contributes to the maintenance, survival, and enhanced migratory capacity of the tumor stem cell population (82, 83). TNF-α promotes the expression of carcinogenic CCL5 variants in breast cancer cells, instigating tumor cell detachment and dissemination (57). Activation of the TLR2/MyD88/NF-κB pathway elevates the autocrine secretion of VEGF and MMP9 in MDA-MB-231 cells, recognized as pivotal factors for breast cancer cell invasion and adhesion (27). The inhibition of NF-κB significantly reduces the invasiveness of MDA-MB-231 cells (84, 85). NF-κB inhibition profoundly diminishes the invasiveness of MDA-MB-231 cells (86, 87).

The MyD88 pathway also mediates immune responses that impact breast cancer progression. For example, Astragalus polysaccharide (APS) may regulate host immunity and exert anti-tumor effects by activating the TLR4-mediated MyD88-dependent signaling pathway (17). A Arazyme activates TLR4-MyD88-TRIF, which reduces primary and metastatic tumor development in the 4T1 murine breast cancer model, thus extending survival (88). Inhibition of proprotein convertases 1/3 steers NR8383 macrophages toward an M1 activation phenotype mainly via TLR4/MyD88 signaling, leading to the secretion of factors that attract immature T helper lymphocytes and promote cytotoxic responses (89). Polysaccharide peptide (PSP) acts by upregulating the TLR4-TIRAP/MAL-MyD88 signaling pathway in peripheral blood mononuclear cells (PBMCs) from breast cancer patients, inducing the secretion of IL-12, IL-6, and TNF-α, thus serving as an immune adjuvant (90). Activation of the TLR5/MyD88/NME3/NFκB signaling pathway enhances host immunity, enhances the clearance of tumor xenografts, and potentially augments the effectiveness of immunotherapy, prolonging survival in breast cancer patients (91). Stimulation of TLR8 ligands through the MyD88/IRAK4/TRAF6/p38 signaling pathway reverses the suppressive function of CD4^+^ CD25^+^ Treg cells, eliminating the inhibitory effect of BTIL31 γδ1 Treg cells on T cell proliferation and function, as well as dendritic cell maturation and function (92).

Indeed, various immune effects have been observed in other cancers as well. Our previous research demonstrated that TJ-M2010-2 successfully mitigated inflammation induced by AOM/DSS, thereby altering the tumor microenvironment and reducing the incidence of colorectal cancer from 100% to 0% (11). MyD88 plays a critical role in tumor-derived exosome-mediated MDSC expansion and subsequent tumor metastasis (93). Furthermore, Pixatimod (PG545), a novel clinical-stage immune modulator, amplifies T cell infiltration when combined with anti-PD-1 therapy through the MyD88-dependent TLR9 pathway. Simultaneously, it inhibits the infiltration of tumor-associated macrophages (TAMs) and activates dendritic cells (DCs), thereby stimulating natural killer (NK) cells (94). IL-33 has the capacity to promote the differentiation and maturation of DC cells via the MyD88 pathway. This results in upregulated tumor immunity in CD8^+^T cells and NK cells while inhibiting the proliferation of lung cancer cells (95).

The multifaceted influence of MyD88 on the tumor immune microenvironment involves the orchestration of multiple cytokines and intricate signaling pathways. A comprehensive comprehension of the role of MyD88 in shaping the tumor microenvironment greatly enhances our understanding of breast cancer development mechanisms, and it offers a strong theoretical foundation for the development of innovative therapeutic strategies. It is imperative that further research endeavors are undertaken to elucidate the intricate mechanisms through which MyD88 exerts its influence on the tumor immune microenvironment and to explore its potential applications in the development of novel treatments.

## Conclusions and prospects

MyD88 assumes multifaceted, pivotal roles in the landscape of breast cancer, influencing diverse aspects such as tumor development, drug resistance, stem cell properties, and the intricacies of the immune microenvironment. A comprehensive understanding of the mechanisms underpinning MyD88’s actions in breast cancer not only unravels the molecular complexities governing tumor progression but also unveils fresh perspectives and potential targets for shaping breast cancer treatment strategies. MyD88 emerges as a valuable asset in prognosis assessment, offering guidance for therapeutic choices and the development of innovative treatment modalities. Nonetheless, there exists a need for further research to unveil the precise workings of MyD88 and translate its applications into clinical practice, with the ultimate aim of achieving more precise and personalized approaches to managing breast cancer.

## Author contributions

HZ: Conceptualization, Project administration, Writing – original draft. XW: Funding acquisition, Project administration, Resources, Writing – original draft. LG: Investigation, Methodology, Writing – original draft. JL: Conceptualization, Funding acquisition, Project administration, Writing – original draft, Writing – review & editing.

## References

[1] Siegel RL Miller KD Wagle NS Jemal A. Cancer statistics, 2023. CA Cancer J Clin (2023) 73(1):17–48. doi: 10.3322/caac.21763 36633525

[2] Sung H Ferlay J Siegel RL Laversanne M Soerjomataram I Jemal A. Global cancer statistics 2020: GLOBOCAN estimates of incidence and mortality worldwide for 36 cancers in 185 countries. CA Cancer J Clin (2021) 71(3):209–49. doi: 10.3322/caac.21660 33538338

[3] Harbeck N Gnant M. Breast cancer. Lancet (2017) 389(10074):1134–50. doi: 10.1016/S0140-6736(16)31891-8 27865536

[4] Li CI Daling JR Malone KE. Incidence of invasive breast cancer by hormone receptor status from 1992 to 1998. J Clin Oncol (2003) 21(1):28–34. doi: 10.1200/JCO.2003.03.088 12506166

[5] von Bernuth H Picard C Jin Z Pankla R Xiao H Ku CL. Pyogenic bacterial infections in humans with MyD88 deficiency. Science (2008) 321(5889):691–6. doi: 10.1126/science.1158298 PMC2688396 18669862

[6] Serbina NV Kuziel W Flavell R Akira S Rollins B Pamer EG. Sequential MyD88-independent and -dependent activation of innate immune responses to intracellular bacterial infection. Immunity (2003) 19(6):891–901. doi: 10.1016/s1074-7613(03)00330-3 14670305

[7] Chen H Jiang Z. The essential adaptors of innate immune signaling. Protein Cell (2013) 4(1):27–39. doi: 10.1007/s13238-012-2063-0 22996173 PMC4875439

[8] Xiang F Fan Y Ni Z Liu Q Zhu Z Chen Z. Ursolic acid reverses the chemoresistance of breast cancer cells to paclitaxel by targeting miRNA-149-5p/myD88. Front Oncol (2019) 9:501. doi: 10.3389/fonc.2019.00501 31259152 PMC6587017

[9] Bohnert KR Goli P Roy A Sharma AK Xiong G Gallot YS. The toll-like receptor/myD88/XBP1 signaling axis mediates skeletal muscle wasting during cancer cachexia. Mol Cell Biol (2019) 39(15):e00184-19. doi: 10.1128/MCB.00184-19 31138662 PMC6639248

[10] Delitto D Delitto AE DiVita BB Pham K Han S Hartlage ER. Human pancreatic cancer cells induce a myD88-dependent stromal response to promote a tumor-tolerant immune microenvironment. Cancer Res (2017) 77(3):672–83. doi: 10.1158/0008-5472.CAN-16-1765 PMC5290036 27864347

[11] Xie L Jiang FC Zhang LM He WT Liu JH Li MQ. Targeting of myD88 homodimerization by novel synthetic inhibitor TJ-M2010-5 in preventing colitis-associated colorectal cancer. J Natl Cancer Inst (2016) 108(4):djv364. doi: 10.1093/jnci/djv364 26712311

[12] Park GS Kim JH. LPS up-regulates ICAM-1 expression in breast cancer cells by stimulating a myD88-BLT2-ERK-linked cascade, which promotes adhesion to monocytes. Mol Cells (2015) 38(9):821–8. doi: 10.14348/molcells.2015.0174 PMC4588726 26299331

[13] Koch AT Love-Homan L Espinosa-Cotton M Stanam A Simons AL. MyD88-dependent signaling decreases the antitumor efficacy of epidermal growth factor receptor inhibition in head and neck cancer cells. Cancer Res (2015) 75(8):1657–67. doi: 10.1158/0008-5472.CAN-14-2061 PMC4401635 25712126

[14] Scheeren FA Kuo AH van Weele LJ Cai S Glykofridis I Sikandar SS. A cell-intrinsic role for TLR2-MYD88 in intestinal and breast epithelia and oncogenesis. . Nat Cell Biol (2014) 16(12):1238–48. doi: 10.1038/ncb3058 25362351

[15] Jia RJ Cao L Zhang L Jing W Chen R Zhu MH. Enhanced myeloid differentiation factor 88 promotes tumor metastasis *via* induction of epithelial-mesenchymal transition in human hepatocellular carcinoma. Cell Death Dis (2014) 5:e1103. doi: 10.1038/cddis.2014.71 24603331 PMC3973199

[16] Husemann Y Geigl JB Schubert F Musiani P Meyer M Burghart E. Systemic spread is an early step in breast cancer. Cancer Cell (2008) 13(1):58–68. doi: 10.1016/j.ccr.2007.12.003 18167340

[17] Zhou L Liu Z Wang Z Yu S Long T Zhou X. Astragalus polysaccharides exerts immunomodulatory effects *via* TLR4-mediated MyD88-dependent signaling pathway in *vitro* and in vivo. Sci Rep (2017) 7:44822. doi: 10.1038/srep44822 28303957 PMC5355992

[18] Wu K Zhang H Fu Y Zhu Y Kong L Chen L. TLR4/MyD88 signaling determines the metastatic potential of breast cancer cells. Mol Med Rep (2018) 18(3):3411–20. doi: 10.3892/mmr.2018.9326 PMC6102647 30066873

[19] Chen X Zhao F Zhang H Zhu Y Wu K Tan G. Significance of TLR4/MyD88 expression in breast cancer. Int J Clin Exp Pathol (2015) 8(6):7034–9. PMC4525929 26261595

[20] Xiang F Ni Z Zhan Y Kong Q Xu J Jiang J. Increased expression of MyD88 and association with paclitaxel resistance in breast cancer. Tumour Biol (2016) 37(5):6017–25. doi: 10.1007/s13277-015-4436-5 26596839

[21] Ma FJ Liu ZB Hu X Ling H Li S Wu J. Prognostic value of myeloid differentiation primary response 88 and Toll-like receptor 4 in breast cancer patients. PloS One (2014) 9(10):e111639. doi: 10.1371/journal.pone.0111639 25360699 PMC4216121

[22] Zandi Z Kashani B Poursani EM Bashash D Kabuli M Momeny M. TLR4 blockade using TAK-242 suppresses ovarian and breast cancer cells invasion through the inhibition of extracellular matrix degradation and epithelial-mesenchymal transition. Eur J Pharmacol (2019) 853:256–63. doi: 10.1016/j.ejphar.2019.03.046 30930249

[23] Wang EL Qian ZR Nakasono M Tanahashi T Yoshimoto K Bando Y. High expression of Toll-like receptor 4/myeloid differentiation factor 88 signals correlates with poor prognosis in colorectal cancer. Br J Cancer (2010) 102(5):908–15. doi: 10.1038/sj.bjc.6605558 PMC2833250 20145615

[24] Szajnik M Szczepanski MJ Czystowska M Elishaev E Mandapathil M Nowak-Markwitz E. TLR4 signaling induced by lipopolysaccharide or paclitaxel regulates tumor survival and chemoresistance in ovarian cancer. Oncogene (2009) 28(49):4353–63. doi: 10.1038/onc.2009.289 PMC2794996 19826413

[25] Liang B Chen R Wang T Cao L Liu Y Yin F. Myeloid differentiation factor 88 promotes growth and metastasis of human hepatocellular carcinoma. Clin Cancer Res (2013) 19(11):2905–16. doi: 10.1158/1078-0432.CCR-12-1245 23549880

[26] Ochi A Nguyen AH Bedrosian AS Mushlin HM Zarbakhsh S Barilla R. MyD88 inhibition amplifies dendritic cell capacity to promote pancreatic carcinogenesis *via* Th2 cells. J Exp Med (2012) 209(9):1671–87. doi: 10.1084/jem.20111706 PMC3428946 22908323

[27] Xie W Wang Y Huang Y Yang H Wang J Hu Z. Toll-like receptor 2 mediates invasion *via* activating NF-kappaB in MDA-MB-231 breast cancer cells. Biochem Biophys Res Commun (2009) 379(4):1027–32. doi: 10.1016/j.bbrc.2009.01.009 19141294

[28] Conti L Lanzardo S Arigoni M Antonazzo R Radaelli E Cantarella D. The noninflammatory role of high mobility group box 1/Toll-like receptor 2 axis in the self-renewal of mammary cancer stem cells. FASEB J (2013) 27(12):4731–44. doi: 10.1096/fj.13-230201 23970797

[29] Romieu-Mourez R Landesman-Bollag E Seldin DC Traish AM Mercurio F Sonenshein GE. Roles of IKK kinases and protein kinase CK2 in activation of nuclear factor-kappaB in breast cancer. Cancer Res (2001) 61(9):3810–8. 11325857

[30] Sovak MA Bellas RE Kim DW Zanieski GJ Rogers AE Traish AM. Aberrant nuclear factor-kappaB/Rel expression and the pathogenesis of breast cancer. J Clin Invest (1997) 100(12):2952–60. doi: 10.1172/JCI119848 PMC508506 9399940

[31] Romieu-Mourez R Landesman-Bollag E Seldin DC Sonenshein GE. Protein kinase CK2 promotes aberrant activation of nuclear factor-kappaB, transformed phenotype, and survival of breast cancer cells. Cancer Res (2002) 62(22):6770–8. 12438279

[32] Booy EP McRae EK Koul A Lin F McKenna SA. The long non-coding RNA BC200 (BCYRN1) is critical for cancer cell survival and proliferation. Mol Cancer (2017) 16(1):109. doi: 10.1186/s12943-017-0679-7 28651607 PMC5483959

[33] Liu JH Chen C Li ZY Zou ZM Gao DC Zhang X. The MyD88 inhibitor TJ-M2010-2 suppresses proliferation, migration and invasion of breast cancer cells by regulating MyD88/GSK-3beta and MyD88/NF-kappaB signalling pathways. Exp Cell Res (2020) 394(2):112157. doi: 10.1016/j.yexcr.2020.112157 32610185

[34] Olson MA Lee MS Kissner TL Alam S Waugh DS Saikh KU. Discovery of small molecule inhibitors of MyD88-dependent signaling pathways using a computational screen. Sci Rep (2015) 5:14246. doi: 10.1038/srep14246 26381092 PMC4585646

[35] Liu X Yao JJ Chen Z Lei W Duan R Yao Z. Lipopolysaccharide sensitizes the therapeutic response of breast cancer to IAP antagonist. Front Immunol (2022) 13:906357. doi: 10.3389/fimmu.2022.906357 36119107 PMC9471085

[36] Egunsola AT Zawislak CL Akuffo AA Chalmers SA Ewer JC Vail CM. Growth, metastasis, and expression of CCL2 and CCL5 by murine mammary carcinomas are dependent upon Myd88. Cell Immunol (2012) 272(2):220–9. doi: 10.1016/j.cellimm.2011.10.008 PMC3244535 22088941

[37] Singh A Devkar R Basu A. Myeloid differentiation primary response 88-cyclin D1 signaling in breast cancer cells regulates toll-like receptor 3-mediated cell proliferation. Front Oncol (2020) 10:1780. doi: 10.3389/fonc.2020.01780 33072559 PMC7531238

[38] Holleman A Chung I Olsen RR Kwak B Mizokami A Saijo N. miR-135a contributes to paclitaxel resistance in tumor cells both *in vitro* and *in vivo* . Oncogene (2011) 30(43):4386–98. doi: 10.1038/onc.2011.148 PMC3572709 21552288

[39] Baldwin AS. Control of oncogenesis and cancer therapy resistance by the transcription factor NF-kappaB. J Clin Invest (2001) 107(3):241–6. doi: 10.1172/JCI11991 PMC199203 11160144

[40] Chang H Wang Y Yin X Liu X Xuan H. Ethanol extract of propolis and its constituent caffeic acid phenethyl ester inhibit breast cancer cells proliferation in inflammatory microenvironment by inhibiting TLR4 signal pathway and inducing apoptosis and autophagy. BMC Complement Altern Med (2017) 17(1):471. doi: 10.1186/s12906-017-1984-9 28950845 PMC5615448

[41] Klein CA. Cancer progression and the invisible phase of metastatic colonization. Nat Rev Cancer (2020) 20(11):681–94. doi: 10.1038/s41568-020-00300-6 33024261

[42] Roessler S Jia HL Budhu A Forgues M Ye QH Lee JS. A unique metastasis gene signature enables prediction of tumor relapse in early-stage hepatocellular carcinoma patients. Cancer Res (2010) 70(24):10202–12. doi: 10.1158/0008-5472.CAN-10-2607 PMC3064515 21159642

[43] Karin M. Nuclear factor-kappaB in cancer development and progression. Nature (2006) 441(7092):431–6. doi: 10.1038/nature04870 16724054

[44] Karin M Greten FR. NF-kappaB: linking inflammation and immunity to cancer development and progression. Nat Rev Immunol (2005) 5(10):749–59. doi: 10.1038/nri1703 16175180

[45] Huber MA Azoitei N Baumann B Grunert S Sommer A Pehamberger H. NF-kappaB is essential for epithelial-mesenchymal transition and metastasis in a model of breast cancer progression. J Clin Invest (2004) 114(4):569–81. doi: 10.1172/JCI21358 PMC503772 15314694

[46] Chalmers SA Eidelman AS Ewer JC Ricca JM Serrano A Tucker KC. A role for HMGB1, HSP60 and Myd88 in growth of murine mammary carcinoma in vitro. Cell Immunol (2013) 282(2):136–45. doi: 10.1016/j.cellimm.2013.04.014 PMC3706557 23770722

[47] Sheng Y Hu R Zhang Y Luo W. MicroRNA-4317 predicts the prognosis of breast cancer and inhibits tumor cell proliferation, migration, and invasion. Clin Exp Med (2020) 20(3):417–25. doi: 10.1007/s10238-020-00625-4 32279128

[48] Zheng X Zhao Y Jia Y Shao D Zhang F Sun M. Biomimetic co-assembled nanodrug of doxorubicin and berberine suppresses chemotherapy-exacerbated breast cancer metastasis. Biomaterials (2021) 271:120716. doi: 10.1016/j.biomaterials.2021.120716 33621894

[49] Bourguignon LY Wong G Earle CA Xia W. Interaction of low molecular weight hyaluronan with CD44 and toll-like receptors promotes the actin filament-associated protein 110-actin binding and MyD88-NFkappaB signaling leading to proinflammatory cytokine/chemokine production and breast tumor invasion. Cytoskeleton (Hoboken) (2011) 68(12):671–93. doi: 10.1002/cm.20544 PMC3240717 22031535

[50] Park GS Kim JH. Myeloid differentiation primary response gene 88-leukotriene B4 receptor 2 cascade mediates lipopolysaccharide-potentiated invasiveness of breast cancer cells. Oncotarget (2015) 6(8):5749–59. doi: 10.18632/oncotarget.3304 PMC4467399 25691060

[51] Haghjooy-Javanmard S Ghasemi A Laher I Zarrin B Dana N Vaseghi G. Influence of morphine on TLR4/ NF-kB signaling pathway of MCF-7 cells. Bratisl Lek Listy (2018) 119(4):229–33. doi: 10.4149/BLL_2018_043 30113863

[52] Andela VB Schwarz EM Puzas JE O'Keefe RJ Rosier RN. Tumor metastasis and the reciprocal regulation of prometastatic and antimetastatic factors by nuclear factor kappaB. Cancer Res (2000) 60(23):6557–62. 11118032

[53] Pan S Guan Y Ma Y Cui Q Tang Z Li J. Advanced glycation end products correlate with breast cancer metastasis by activating RAGE/TLR4 signaling. BMJ Open Diabetes Res Care (2022) 10(2):e002697. doi: 10.1136/bmjdrc-2021-002697 PMC8961114 35346972

[54] Yang H Wang B Wang T Xu L He C Wen H. Toll-like receptor 4 prompts human breast cancer cells invasiveness *via* lipopolysaccharide stimulation and is overexpressed in patients with lymph node metastasis. PloS One (2014) 9(10):e109980. doi: 10.1371/journal.pone.0109980 25299052 PMC4192367

[55] De Craene B Berx G. Regulatory networks defining EMT during cancer initiation and progression. Nat Rev Cancer (2013) 13(2):97–110. doi: 10.1038/nrc3447 23344542

[56] Huber MA Kraut N Beug H. Molecular requirements for epithelial-mesenchymal transition during tumor progression. Curr Opin Cell Biol (2005) 17(5):548–58. doi: 10.1016/j.ceb.2005.08.001 16098727

[57] Nieto MA Huang RY Jackson RA Thiery JP. Emt: 2016. Cell (2016) 166(1):21–45. doi: 10.1016/j.cell.2016.06.028 27368099

[58] Chua HL Bhat-Nakshatri P Clare SE Morimiya A Badve S Nakshatri H. NF-kappaB represses E-cadherin expression and enhances epithelial to mesenchymal transition of mammary epithelial cells: potential involvement of ZEB-1 and ZEB-2. Oncogene (2007) 26(5):711–24. doi: 10.1038/sj.onc.1209808 16862183

[59] Huang T Chen Z Fang L. Curcumin inhibits LPS-induced EMT through downregulation of NF-kappaB-Snail signaling in breast cancer cells. Oncol Rep (2013) 29(1):117–24. doi: 10.3892/or.2012.2080 23076367

[60] Chen MC Chang WW Kuan YD Lin ST Hsu HC Lee CH. Resveratrol inhibits LPS-induced epithelial-mesenchymal transition in mouse melanoma model. Innate Immun (2012) 18(5):685–93. doi: 10.1177/1753425912436589 22344225

[61] Liu JH He L Zou ZM Ding ZC Zhang X Wang H. A novel inhibitor of homodimerization targeting myD88 ameliorates renal interstitial fibrosis by counteracting TGF-beta1-induced EMT in vivo and in vitro. Kidney Blood Press Res (2018) 43(5):1677–87. doi: 10.1159/000494745 30380557

[62] Zhang LM Liu JH Xue CB Li MQ Xing S Zhang X. Pharmacological inhibition of MyD88 homodimerization counteracts renal ischemia reperfusion-induced progressive renal injury *in vivo* and *in vitro* . Sci Rep (2016) 6:26954. doi: 10.1038/srep26954 27246399 PMC4887891

[63] Nussinov R Tsai CJ Jang H. Anticancer drug resistance: An update and perspective. Drug Resist Update (2021) 59:100796. doi: 10.1016/j.drup.2021.100796 PMC8810687 34953682

[64] Zhang W Zheng X Meng T You H Dong Y Xing J. SET protein overexpression contributes to paclitaxel resistance in MCF-7/S cells through PI3K/Akt pathway. J Drug Target (2017) 25(3):255–63. doi: 10.1080/1061186X.2016.1245307 27718638

[65] Poh Yen K Stanslas J Zhang T Li H Wang X Kok Meng C. Synthesis of small molecules targeting paclitaxel-induced MyD88 expression in triple-negative breast cancer cell lines. Bioorg Med Chem (2021) 49:116442. doi: 10.1016/j.bmc.2021.116442 34600241

[66] Zhang L Chen T Yan L Xu H Wang Y Li Y. MiR-155-3p acts as a tumor suppressor and reverses paclitaxel resistance *via* negative regulation of MYD88 in human breast cancer. Gene (2019) 700:85–95. doi: 10.1016/j.gene.2019.02.066 30878390

[67] Saha S Mukherjee S Khan P Kajal K Mazumdar M Manna A. Aspirin suppresses the acquisition of chemoresistance in breast cancer by disrupting an NFkappaB-IL6 signaling axis responsible for the generation of cancer stem cells. Cancer Res (2016) 76(7):2000–12. doi: 10.1158/0008-5472.CAN-15-1360 26842876

[68] Korkaya H Kim GI Davis A Malik F Henry NL Ithimakin S. Activation of an IL6 inflammatory loop mediates trastuzumab resistance in HER2+ breast cancer by expanding the cancer stem cell population. Mol Cell (2012) 47(4):570–84. doi: 10.1016/j.molcel.2012.06.014 PMC3432419 22819326

[69] Burnett JP Lim G Li Y Shah RB Lim R Paholak HJ. Sulforaphane enhances the anticancer activity of taxanes against triple negative breast cancer by killing cancer stem cells. Cancer Lett (2017) 394:52–64. doi: 10.1016/j.canlet.2017.02.023 28254410 PMC8892390

[70] Cao N Li S Wang Z Ahmed KM Degnan ME Fan M. NF-kappaB-mediated HER2 overexpression in radiation-adaptive resistance. Radiat Res (2009) 171(1):9–21. doi: 10.1667/RR1472.1 19138055 PMC2659759

[71] Edwardson DW Boudreau J Mapletoft J Lanner C Kovala AT Parissenti AM. Inflammatory cytokine production in tumor cells upon chemotherapy drug exposure or upon selection for drug resistance. PloS One (2017) 12(9):e0183662. doi: 10.1371/journal.pone.0183662 28915246 PMC5600395

[72] Badve S Nakshatri H. Breast-cancer stem cells-beyond semantics. Lancet Oncol (2012) 13(1):e43–8. doi: 10.1016/S1470-2045(11)70191-7 22225725

[73] Colavito SA Zou MR Yan Q Nguyen DX Stern DF. Significance of glioma-associated oncogene homolog 1 (GLI1) expression in claudin-low breast cancer and crosstalk with the nuclear factor kappa-light-chain-enhancer of activated B cells (NFkappaB) pathway. Breast Cancer Res (2014) 16(5):444. doi: 10.1186/s13058-014-0444-4 25252859 PMC4303124

[74] Johnson DE O'Keefe RA Grandis JR. Targeting the IL-6/JAK/STAT3 signalling axis in cancer. Nat Rev Clin Oncol (2018) 15(4):234–48. doi: 10.1038/nrclinonc.2018.8 PMC5858971 29405201

[75] Waugh DJ Wilson C. The interleukin-8 pathway in cancer. Clin Cancer Res (2008) 14(21):6735–41. doi: 10.1158/1078-0432.CCR-07-4843 18980965

[76] Zhen X Choi HS Kim JH Kim SL Liu R Yun BS. Machilin D, a lignin derived from saururus chinensis, suppresses breast cancer stem cells and inhibits NF-kappaB signaling. Biomolecules (2020) 10(2):245. doi: 10.3390/biom10020245 32033472 PMC7072518

[77] Pitt JM Marabelle A Eggermont A Soria JC Kroemer G Zitvogel L. Targeting the tumor microenvironment: removing obstruction to anticancer immune responses and immunotherapy. Ann Oncol (2016) 27(8):1482–92. doi: 10.1093/annonc/mdw168 27069014

[78] Zahn LM. Effects of the tumor microenvironment. Science (2017) 355(6332):1386–8. doi: 10.1126/science.355.6332.1386-l 28360308

[79] Joyce JA Fearon DT. T cell exclusion, immune privilege, and the tumor microenvironment. Science (2015) 348(6230):74–80. doi: 10.1126/science.aaa6204 25838376

[80] Chow A Zhou W Liu L Fong MY Champer J Van Haute D. Macrophage immunomodulation by breast cancer-derived exosomes requires Toll-like receptor 2-mediated activation of NF-κB. Sci Rep (2014) 4(1):5750. doi: 10.1038/srep05750 25034888 PMC4102923

[81] Korkaya H Liu S Wicha MS. Regulation of cancer stem cells by cytokine networks: attacking cancer's inflammatory roots. Clin Cancer Res (2011) 17(19):6125–9. doi: 10.1158/1078-0432.CCR-10-2743 PMC3312242 21685479

[82] Liu Z Bandyopadhyay A Nichols RW Wang L Hinck AP Wang S. Blockade of autocrine TGF-beta signaling inhibits stem cell phenotype, survival, and metastasis of murine breast cancer cells. J Stem Cell Res Ther (2012) 2(1):1–8. doi: 10.4172/2157-7633.1000116 23482850 PMC3593047

[83] Zhao Z Hao D Wang L Li J Meng Y Li P. CtBP promotes metastasis of breast cancer through repressing cholesterol and activating TGF-beta signaling. Oncogene (2019) 38(12):2076–91. doi: 10.1038/s41388-018-0570-z 30442980

[84] Omene C Bandera EV. Anti-VEGF therapy - a role in obesity-related breast cancer. Nat Rev Endocrinol (2018) 14(6):329–30. doi: 10.1038/s41574-018-0021-5 PMC6497165 29695750

[85] Sansone P Storci G Tavolari S Guarnieri T Giovannini C Taffurelli M. IL-6 triggers Malignant features in mammospheres from human ductal breast carcinoma and normal mammary gland. J Clin Invest (2007) 117(12):3988–4002. doi: 10.1172/JCI32533 18060036 PMC2096439

[86] Sung B Pandey MK Nakajima Y Nishida H Konishi T Chaturvedi MM. Identification of a novel blocker of IkappaBalpha kinase activation that enhances apoptosis and inhibits proliferation and invasion by suppressing nuclear factor-kappaB. Mol Cancer Ther (2008) 7(1):191–201. doi: 10.1158/1535-7163.MCT-07-0406 18202022

[87] Park BK Zhang H Zeng Q Dai J Keller ET Giordano T. NF-kappaB in breast cancer cells promotes osteolytic bone metastasis by inducing osteoclastogenesis *via* GM-CSF. Nat Med (2007) 13(1):62–9. doi: 10.1038/nm1519 17159986

[88] Pereira FV Melo AC de Melo FM Mourao-Sa D Silva P Berzaghi R. TLR4-mediated immunomodulatory properties of the bacterial metalloprotease arazyme in preclinical tumor models. Oncoimmunology (2016) 5(7):e1178420. doi: 10.1080/2162402X.2016.1178420 27622031 PMC5006932

[89] Duhamel M Rodet F Delhem N Vanden Abeele F Kobeissy F Nataf S. Molecular consequences of proprotein convertase 1/3 (PC1/3) inhibition in macrophages for application to cancer immunotherapy: A proteomic study. Mol Cell Proteomics (2015) 14(11):2857–77. doi: 10.1074/mcp.M115.052480 PMC4638031 26330543

[90] Wang J Dong B Tan Y Yu S Bao YX. A study on the immunomodulation of polysaccharopeptide through the TLR4-TIRAP/MAL-MyD88 signaling pathway in PBMCs from breast cancer patients. Immunopharmacol Immunotoxicol (2013) 35(4):497–504. doi: 10.3109/08923973.2013.805764 23802631

[91] Zhang W Xiong Z Wei T Li Q Tan Y Ling L. Nuclear factor 90 promotes angiogenesis by regulating HIF-1alpha/VEGF-A expression through the PI3K/Akt signaling pathway in human cervical cancer. Cell Death Dis (2018) 9(3):276. doi: 10.1038/s41419-018-0334-2 29449553 PMC5833414

[92] Peng G Wang HY Peng W Kiniwa Y Seo KH Wang RF. Tumor-infiltrating gammadelta T cells suppress T and dendritic cell function *via* mechanisms controlled by a unique toll-like receptor signaling pathway. Immunity (2007) 27(2):334–48. doi: 10.1016/j.immuni.2007.05.020 17656116

[93] Liu Y Xiang X Zhuang X Zhang S Liu C Cheng Z. Contribution of MyD88 to the tumor exosome-mediated induction of myeloid derived suppressor cells. Am J Pathol (2010) 176(5):2490–9. doi: 10.2353/ajpath.2010.090777 PMC2861113 20348242

[94] Hammond E Haynes NM Cullinane C Brennan TV Bampton D Handley P. Immunomodulatory activities of pixatimod: emerging nonclinical and clinical data, and its potential utility in combination with PD-1 inhibitors. J Immunother Cancer (2018) 6(1):54. doi: 10.1186/s40425-018-0363-5 29898788 PMC6000956

[95] Xu L Zheng Y Wang J Xu Y Xie Y Yang ZP. IL33 activates CD8+T and NK cells through MyD88 pathway to suppress the lung cancer cell growth in mice. Biotechnol Lett (2020) 42(7):1113–21. doi: 10.1007/s10529-020-02815-2 32140881

